# 

**Published:** 1886-07

**Authors:** 


### CONTENTS:

ARTICLE I—Recent Progress in Dentistry. By William Herbert Rollins.......... 97

" II—Pocket Disease of the Alveolus. By J. N. Farrar, M. D., D. D. S..... 104

" III—A Visit to Foreign Dental Schools and other Observations. By A. W. Harlan, M. D., D. D. S.......... 110

" IV—Death from Chloroform.......... 118

" V—Irregularities of the Teeth and their Treatment. By F. E. Howard, M. D 120

" VI—The Treatment of Pulpless and Gaseous Teeth. By J. Taft.......... 124

" VII—Advice to Those who Wear Artificial Teeth.......... 127

" VIII—The Maryland Dental Law. By Richard Grady, D D. S.......... 129

" IX—A Peculiar Case of Neuralgia of the Trigeminus. By H. Hamecher, D.D.S 131

Southern Dental Association.......... 135

### EDITORIAL, ETC.

The Source of Scarlet Fever.......... 137

### BIBLIOGRAPHICAL.

Diseases of the Digestive Organs in Infancy and Childhood.......... 139
Facial Prosthesis.......... 140
The Therapeutics of High Temperatures in Young Children.......... 140
Reproduction of Tissue by Skin Grafting.......... 140
Atmospheric Purification.......... 140
Address before the Alumni Association of the Department of Medicine and Surgery, of the University of Michigan.......... 140

### MONTHLY SUMMARY.

How to Become Thin.......... 141
Professor Huxley on Smoking.......... 142
Distinguished Honor.......... 142
Final Results of Operation for Cancer of the Lip.......... 143
Fun Among the Doctors.......... 144

